# Alarm Fatigue in the Emergency Department: A Multicenter, Mixed-Method Study of Monitor Alarms

**DOI:** 10.1016/j.acepjo.2025.100077

**Published:** 2025-02-27

**Authors:** Sebastian Johansson, Erika Johannesson, Erika Hörlin, Jens Wretborn

**Affiliations:** Departments of Emergency Medicine and Biomedical and Clinical Sciences, Linköping University, Linköping, Sweden

**Keywords:** alarm fatigue, monitor alarms, false alarm, alarm management

## Abstract

**Objectives:**

Describe the frequency and pattern of monitor alarms in the emergency department (ED), in combination with exploring the staff’s experience of alarms and alarm fatigue.

**Methods:**

This was a multicenter, mixed-method study of observational monitor alarms and semistructured staff interviews with inductive qualitative content analysis from 3 EDs in Sweden. The primary measures were alarm frequency and confirmation times. Quantile regression was used to analyze the association between alarms and confirmation times.

**Results:**

In total, 396,011 alarms were registered during the data collection period, or 1 alarm every 30 seconds in the urban and academic centers and every 120 seconds in the rural hospital, on average. Median confirmation times were 11 seconds for high severity alarms (IQR, 5-33) and 132 seconds for low severity alarms (IQR, 15-878). This increased by 1 second when alarms per hour increased by 128 (95% CI, 67-1000; *P* = .03) and 4.8 (95% CI, 3.1-11.6; *P* = .001) for high and low severity alarms, respectively. The content analysis from 20 interviews revealed 3 main aspects that influenced alarm management in the ED, with implications on alarm fatigue: unclear or broad indications for monitoring relying on health care staff *knowledge and experience*; physical layout, alarm responsibility, and workload in the *ED environment;* and finally, monitor and patient factors influencing *alarm analysis*.

**Conclusion:**

ED staff work in an alarm environment prone to alarm fatigue with frequent alarms and several system-related factors that increase the risk of alarm fatigue, which is mitigated by the individual health care worker.

**The Bottom Line**

Continuous monitoring of vital signs is commonly used in the emergency department, but it is unknown how staff manage these alarms. In 3 emergency departments in Sweden, monitoring alarms occurred frequently, once every 30 to 120 seconds on average. Together with broad indications, unclear responsibility, and open spaces, there was a risk of alarm fatigue, desensitizing staff to the alarms. Alarm fatigue may prevent staff from utilizing alarms to identify patient deterioration, causing patient harm. The results from our study may be used to identify and implement interventions to reduce the risk of alarm fatigue in emergency departments.

## 1 Introduction

### 1.1 Background

Technical devices are used for the continuous monitoring of patients’ vital signs (saturation, heart rate, heart rhythm, blood pressure, and respiratory rate) in modern health care systems. Monitoring enables early detection of patient deterioration and the possibility for health care staff to oversee multiple patients at the same time.^1^, ^2^, ^3^ However, the monitors have been shown to cause large volumes of alarms that rarely lead to an intervention.^1^^,^^4^ The high volume of alarms affects the workplace environment and may cause alarm fatigue when staff becomes tired and desensitized to alarms.^3^^,^^5^^,^^6^ This poses a threat to patient care, and the Joint Commission has declared alarms from patient monitoring devices a focus area to improve patient safety.^7^

Although the volume of alarms increases the risk of alarm fatigue, it is the response of the staff that will determine the effect on the delivery of care.^2^ Hence, it is important to understand how staff manage alarms to identify potential solutions to improve patient care. In the intensive care unit (ICU), monitor sounds are part of the environment,^8^ with reports of alarm fatigue and situations of patient harm because of missed alarms.^9^

The emergency department (ED) has been identified as an environment with a high risk of alarm fatigue due to the abundance of alarms in a stressful context with high acuity, time-critical tasks, and conditions.^5^^,^^10^^,^^11^ An observational study from an ED in England found continuous sounding from alarms in 29% of the 93 hours of observation with few associated interventions.^1^ Another study from an academic ED in Finland found 28,000 alarms during a month in their observation unit.^12^ However, no studies have investigated staff's experience of alarms in the ED, an environment with considerable differences in staffing, physical layout, and patient composition compared with the ICU and hospital wards.

### 1.2 Importance

Rapid identification of patient deterioration is an essential part of ED operations, and monitoring devices allow limited staff resources to monitor several patients simultaneously. Investigating the perception and management of alarms in the ED is necessary to understand alarm fatigue and ways to mitigate its negative effect on patient care.

### 1.3 Goals of This Investigation

We investigated the alarm environment in the ED in combination with interviews with ED staff, including physicians, nurses, and assistant nurses, to explore staff’s interaction with and attitudes toward monitor alarms as well as the presence of alarm fatigue.

## 2 Methods

### 2.1 Study Design and Setting

This was a mixed-method study at 3 EDs in Sweden’s Region Östergötland health care system using retrospective observational monitor data and prospective semistructured interviews with ED staff. The study was approved by the Swedish Ethical Review Authority (permit 2023-06396-01).

Sweden publicly funds health care operated by 21 regional health care systems. The 3 EDs in Region Östergötland have a catchment area with approximately 470,000 inhabitants (Table 1). All EDs use the same monitor equipment with similar default alarm thresholds (Supplementary Appendix 1). The equipment is used both in triage and for continuous monitoring, with a monitor at the patient's bedside and 1 central monitor screen next to the working station of the team responsible for the patient. There are no designated staff for these central monitors.

**Table 1:** Emergency department demographics.

Characteristic	Academic tertiary center	Rural community	Urban community
Annual visits, n	51,500	23,000	51,000
Female, n (%)	26,500 (51)	11,700 (51)	25,000 (49)
Arrival by ambulance, n (%)	12,212 (24)	5445 (11)	14,886 (29)
Acuity, n (%)			
1 (high)	2299 (5)	857 (4)	1967 (4)
2	10,924 (21)	4283 (18)	11,852 (23)
3	20,551 (40)	10,250 (44)	22,639 (45)
4 (low)	17,719 (34)	7859 (34)	14,412 (28)
>70 y, n (%)	14,300 (28)	7800 (34)	14,900 (29)
Hospital admissions, n (%)	10,385 (20)	3999 (17)	10,565 (21)
No. of monitors	32	22	31
Length of stay, minutes (median)	205	172	245
Nurse hours per day	170	86	162

There are 3 levels of alarms that make different beeping sounds at the bedside and the central monitor simultaneously. Based on the typology by Ruskin and Heuske-Kras,^5^ these can be separated into *clinical alarms*, which include level 1 and 2 alarms, and *technical alarms* (level 3). *Clinical alarms* are triggered by abnormal vital signs, and level 1 alarms indicate severe deviations, like asystole, ventricular tachycardia, or desaturation/severe hypoxia. Less severe clinical alarms, level 2 alarms, including hypoxia (>80% and <90%) and fast heart rate (>110 and <130 beats per minute), among others (Supplementary Appendix 1). *Technical alarms* trigger when the monitor is unable to register the vital signs due to presumed sensor faults, such as loose cardiac electrodes. Level 1 alarms must be confirmed manually at the bedside monitor, whereas level 2 and 3 alarms will silence automatically if vital signs normalize but still have to be confirmed to resolve.

All 3 EDs use the Rapid Emergency Triage and Treatment System to determine acuity on a scale from 1 to 4. The triage system does not mandate continuous monitoring, but local guidelines imply that acuity 1 and 2 patients (high acuity) should have continuous monitoring until the treating physician has seen the patient. Nurses may change alarm thresholds before the physician sees the patient based on clinical judgment.

### 2.2 Participants

During 5 convenience days in January 2024, physicians, nurses, and nurse assistants were recruited using a purposive sampling strategy, where the researcher approaches potential participants by intentional selection to obtain a representative group of informants.^13^ In this study, we recruited based on role, working experience, and sex (male or female) until thematic saturation.

### 2.3 Quantitative Variables and Statistical Methods

Alarm data from October to November 2023 were extracted from the central alarm server. For each alarm at the 3 EDs, we collected monitor, alarm type, and start and confirmation time. Numbers are presented as percentages, mean with SD, or medians with IQRs. The association between median confirmation time and hour of day was tested using the Kruskal-Wallis test. Quantile regression was used to test the association between the median confirmation time for each alarm type and the number of alarms per hour.

### 2.4 Qualitative Variables and Methods

We designed and tested an interview guide on 6 clinicians to examine the perceived meaning of the questions, as well as their ease of understanding. After adjustments, the final version was tested on a researcher colleague with qualitative methodology experience. None of these clinicians were subsequently in the study. There were 8 main questions in the interview guide (Supplementary Appendix 2). Alarm fatigue was identified either by the interviewee directly or by describing situations where monitor alarms prevented an intended or desired process.

Semistructured interviews were performed by 2 of the authors (EJ and SJ) between January 16 and January 25, 2024, alternating roles as observer and interviewer between interviews. At the end of the interview, the observer had the ability to ask further questions. The interviews were recorded and then transcribed verbatim using the noScribe software. All transcripts were checked in their entirety, corrected by EJ or SJ, and analyzed according to Elo and Kyngäs^14^ using an inductive approach. Initially, all text was read through by EJ and SJ individually, and meaning units, words, or sentences describing a phenomenon were identified. EJ and SJ individually coded the selected meaning units by content, and differences were discussed until a consensus was reached. Subcategories and categories were created by identifying patterns and connections (Table 2). The final abstraction process involved all authors that inductively identified association, interaction, and affinity between categories.

**Table 2:** Example of qualitative analysis.

Quote – meaning unit	Open coding	Subcategory	Category
*“I have been told that everyone who is triaged as acuity 2 and above according to RETTS (Rapid Emergency Triage and Treatment System). So, acuteness 1 or 2 should have (monitoring)...”*	Triage acuity is an indication for monitoring	Monitor indication	Knowledge and experience
*“Maybe it also depends on which team you're placed on. On some teams, I think patients are more likely to be monitored, even though they don't have to. So even patients with acuity 3 and 4 (according to triage) can be connected to a monitor without the needs for it”* - *Why is that?(interviewer)* *“inexperienced and insecurity (among staff)”*	ED teams with inexperienced staff members are more likely to have patients monitored, caused by insecurity in monitoring	Monitor knowledge	

ED, emergency department.

## 3 Results

### 3.1 Monitor Alarms

During October and November 2023, a total of 396,011 alarms were registered, of which 339,961 (85%) were technical alarms, and 42,456 (11%) and 16,885 (4%) were level 1 and 2 clinical alarms, respectively. This corresponds to 1 alarm every 30 seconds in the urban (18 per nurse/h) and tertiary care center (17 per nurse/h) and every 120 seconds in the rural hospital (10 per nurse/h), on average. The median number of clinical alarms per monitor and day was 14 (IQR, 12-16), 15 (IQR, 10-21), and 11 (IQR, 9-20), and technical alarms were 83 (IQR, 56-110; maximum, 158), 89 (IQR, 44-123), and 34 (IQR, 28-55) for the academic, urban, and rural hospital, respectively. The median confirmation times were 11 seconds (IQR, 5-33) and 132 seconds (IQR, 15-878) for level 1 and 2 alarms, respectively.

**Figure 1:**
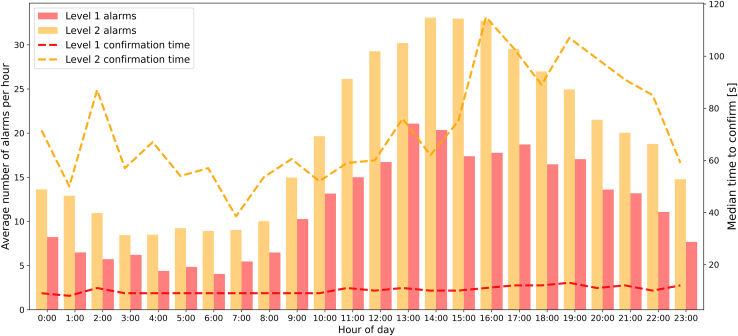
The average number of clinical alarms per hour in relation to the average confirmation time in 1 academic, 1 urban, and 1 rural emergency department in Sweden.

There was a diurnal pattern for clinical alarms, but it was not significantly higher at any particular time (Kurskal-Wallis *P* = .46) (Fig 1). Technical alarms had a similar diurnal pattern ranging from 100 (5 am) to 350 (1-6 pm) alarms per hour. Median confirmation time increased by 1 second when the total number of alarms per hour increased by 128 (95% CI, 67-1000; *P* = .03) and 4.8 (95% CI, 3.1-11.6; *P* = .001) for level 1 and 2 alarms, respectively (Supplementary Appendix 1).

The most frequent clinical alarms were hypoxia, abnormal heart rate, ventricular tachycardia, and abnormal blood pressure (noninvasive) (Table 3). The most frequent technical alarms were those indicating faulty signals for pulse oximetry, cardiac monitoring, and respiratory rate (Supplementary Appendix 1).

**Table 3:** Types of clinical alarms based on monitor assessments.

Alarm type	Rural ED, n (%)	Academic ED, n (%)	Urban ED, n (%)
No. of alarms	8317	22,279	25,454
Hypoxia	4251 (51)	8076 (36)	8460 (33)
Heart frequency	849 (10)	4570 (21)	5969 (23)
Ventricular tachycardia	799 (10)	1968 (9)	2347 (9)
Noninvasive blood pressure	621 (7)	2728 (12)	2439 (10)
Desaturation	472 (6)	1769 (8)	2501 (10)
Other	1325 (16)	3168 (14)	3738 (15)

ED, emergency department.

### 3.2 Interviews

In total, 20 ED staff were interviewed, with half having worked 6 years or more in the ED, and 70% of the participants were female (Table 4). The interviews lasted between 9 and 21 minutes and started with an open question about the participants’ thoughts on monitor alarms in general. All participants associated monitor alarms with frequent beeping, and the majority thought of false alarms, whereas some associated it with a necessity to react.

**Table 4:** Demographic description of the participants in the semistructured interviews.

Profession	Physician	Registered nurse	Assistant nurse
n	6	7	7
Years in the ED, median (range)	7 (1 mo-10 y)	2 (2 mo-25 y)	6 (1 mo-23 y)
Age (y), median (range)	37 (28-43)	35 (26-62)	51 (33-62)
Female, n (%)	3 (50)	4 (57)	7 (100)

ED, emergency department.

**Figure 2:**
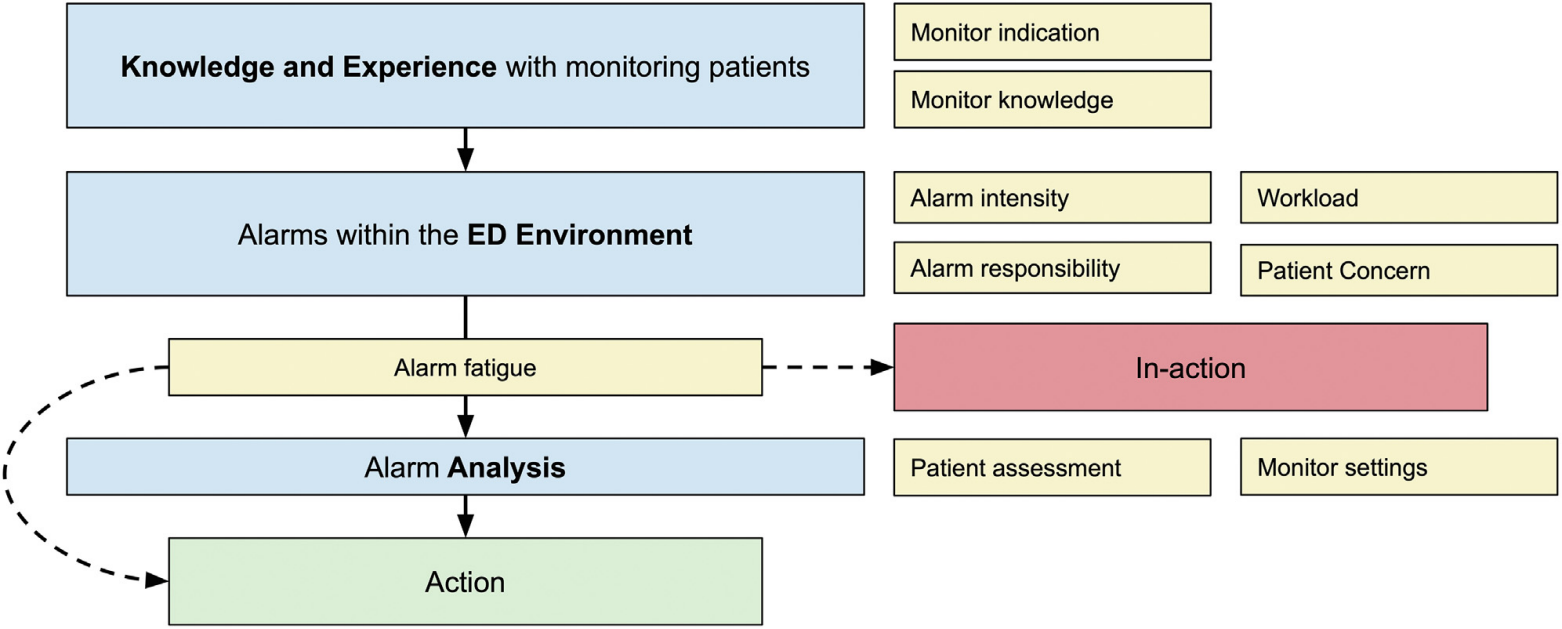
Alarm management in a challenging environment – from indication to action – is based on categories (blue) and subcategories (yellow) from the inductive content analysis. ED, emergency department.

The highest level of abstraction, which encompassed the whole interview material, was summarized as a concept of *“Alarm management in a challenging environment - from indication to action.”* Three main categories, *Knowledge and Experience with monitoring patients*, *Alarms within the ED environment,* and *Alarm Analysis,* and 10 subcategories were identified through the open coding (Fig 2).

### 3.3 Knowledge and Experience with Monitoring Patients

Triage guidelines initiated the decision to start continuous monitoring for many participants. Prior work experience influenced this decision, both to start monitoring patients outside the guidelines and to withhold monitoring.

#### 3.3.1 Monitor indication

Participants experienced a high incidence of unreflected use of monitors, described as following the triage guideline without considering if it was a reasonable indication. Several described situations where monitoring was unnecessary and could be stressful for the patient and did not change care, but some pointed out that it could be a more challenging decision not to monitor or to remove the monitors because of a sense of security.

*“It's pretty easy to have them monitored. It will be a more active decision to remove it.”*

(Resident, 1 year of experience)

#### 3.3.2 Monitor knowledge

Some participants said that more experienced staff saw monitoring more as an adjunct to the clinical assessment compared with inexperienced staff, where monitors are seen as standard procedures. Many participants thought teams with less experienced staff had a tendency to put more patients on monitors without reflecting on the purpose or utility of the monitor.

*“…inexperienced staff that are told that this patient requires monitoring may not reflect on what they want to monitor, or why. They just do it, because the monitors are there.”*

(Nurse, 8 years of experience)

### 3.4 Alarms Within the ED Environment

The environment in the ED was frequently brought up as problematic by participants, and several contributing factors, intrinsic and extrinsic to the monitors, were described. Intrinsic factors included high alarm sensitivity, resulting in high rates of false positives. Extrinsic factors included the workload in the ED, the lack of designated responsibility for the alarms, and concern for patients. Several participants also recognized the benefit of simultaneous patient monitoring.

#### 3.4.1 Workload

Participants described a work environment with frequent alarms, subsequent stress, and constant interruptions in work tasks. The physical design of the ED had a negative impact, with several patients being monitored in the same area, where the beeping sounds could be heard by everyone, even the surrounding teams. Participants also saw a risk of missing alarms when they were not close to the monitors.

*“Sometimes I get mentally exhausted when there is too much beeping. But it's a part of my everyday life. When I started here I was probably more stressed about it…”*

(Registered nurse, 6 years of experience)

#### 3.4.2 Patient concern

Several participants described empathy and concern for patients and their relatives exposed to all the beeping for many hours without knowing why it is beeping and why no one reacts. Several participants mentioned that patients with dementia, in particular, seem to suffer from all the beeping.

*“I think they're lying in their bed wondering what sounds and what the beeping is. Because it is these sounds that make patients worried.”*

(Assistant nurse, 23 years of experience)

#### 3.4.3 Alarm intensity

Most participants experienced a high frequency of alarms, which they linked to the number of acutely ill patients and crowding in the ED, resulting in more alarms and less time for staff to confirm and manage them appropriately. They also noted that false alarms were common, generally attributing this to the monitors being sensitive.

*“We have several patients monitored at the same time here… it will be a lot of alarm because it beeps for everyone… I think I hear more alarms here than I have done at other places.”*

(Resident, 1 year of experience)

#### 3.4.4 Monitoring responsibility

Almost every participant felt that the entire team shares the responsibility for alarms but that it is probably the nurse who has the ultimate responsibility. A few participants thought that it was the physician with the highest medical competence who had the ultimate responsibility but acknowledged the lack of clear guidelines.

*“I think everyone in the team has a responsibility. It can absolutely happen that it can be a lot of beeping when the nurse is not around. The assistant nurse may not react to it and not the physician either because they think it's someone else problem.”*

(Physician, 10 years of experience)

### 3.5 Alarm Analysis

Participants described the process of analyzing the alarm before acting on it, including prior experience, knowledge about the patient, and individual personality traits. Several participants described the traits of alarm fatigue, without mentioning the term per se, as a phenomenon that would preclude the analysis and lead to inaction or bias in the normal analysis process.

#### 3.5.1 Alarm fatigue

The participants described that sometimes staff ignore alarms because they are occupied with other tasks, in particular, during multiple simultaneous alarms or frequent alarms from the same patient. Some of the participants thought this led to delayed response times for clinical alarms, whereas others believed that the staff still reacted rapidly to high severity (level 1) clinical alarms.

*“There is a lot of alarm which leads to ‘the boy who cried wolf.’ You have heard the alarm so many times when it hasn't been anything, so you think it is nothing when the alarm goes off.”*

(Nurse, 8 years of experience)

#### 3.5.2 Patient assessment and monitor settings

Many described the risk of focusing solely on the monitor values and “getting stuck in numbers” and emphasized the importance of combining monitoring values with clinical assessment. Furthermore, it was expressed that the staff's knowledge about the patient's condition, medical history, vital signs in triage, monitor settings, and initial indication affected the tolerance and action related to the alarms, as certain types of alarms were expected for specific conditions. Participants expressed a need to adapt the alarm thresholds to individual patients but noted that this was rarely done, with many false and expected but unnecessary alarms as a result.

*“If you trust your clinical gestalt, this will help you in the assessment whether a patient is getting worse or not, not just looking at the monitor.”*

(Nurse, 8 years of experience)

## 4 Limitations

This study has several limitations. We did not have the ability to quantify the clinical implications of the alarms and could not assess the rate of false or true positive alarms. We did not measure individual nurses’ exposure to alarms, but both staffing and alarms correlated. E.J. and S.J., who performed the interviews, are both working at one of the study sites but did not experience any difference in the interviews between their working ED compared with the other EDs.

## 5 Discussion

In this multicenter, mixed-method study, we show a high volume of alarms in EDs and the complex interaction between the staff and alarms. With 396,011 alarms over 2 months, they are an inherent part of the ED, and staff raised concerns both about their own workplace environment as well as the impact on the patients and their care.

There is a previous report of alarms from a Finnish ED that looked at 8 monitors out of a total of 46 in their ED in their observation area that had 28,000 alarms over 32 days,^12^ which is similar to that of the monitors with the highest alarm rates in our study. Considering the large variation in alarm frequency between monitors across all study EDs, the utilization and location of the monitors likely impacted the overall alarm volume. The staff mentioned the physical design of the ED as a factor that increases the alarm exposure further, with open space solutions affecting surrounding teams. The ED has been shown to have the highest noise levels in the hospital,^15^ and we know from interventions in the ICU that the physical design of units impacts noise levels.^16^ How the physical design of the ED affects and modifies the alarm intensity and noise levels should be further studied.

Previous studies have demonstrated a reduction in cardiac-related alarm frequency between 9% and 50% through the implementation of active threshold adjustments and appropriate skin preparation to prevent electrode faults.^17^^,^^18^ We see the potential for further alarm reduction by improving routines and monitoring protocols for cardiac monitoring, as well as pulse-oximetry and blood pressure. Furthermore, more research is needed to identify patient groups who benefit from continuous monitoring.^19^

The Joint Commission has described alarms from monitoring equipment as one of the most important areas of patient safety for more than a decade, and fatal incidents related to missed alarms have been reported.^3^ Monitoring equipment has become more readily available; however, in our study, many of the basic recommendations from the commission to reduce alarm fatigue have not been implemented or have been unsuccessful. The concept map in this study identifies areas that enable tailored interventions to different aspects of alarm management.

In summary, our results indicate that ED staff work in an alarm environment prone to alarm fatigue with nonspecific indications for monitoring, unclear alarm responsibility, high workload and alarm volumes, and a physical environment that enhances ambient sound. These system-related problems are likely mitigated by the individual health care worker that upholds patient care. Understanding the management of clinical alarms in the ED may provide potential solutions to decrease the risk of alarm fatigue.

## Author Contributions

SJ and EJ conceived the study and designed it together with EH and JW. SJ and EJ conducted and transcribed the interviews and analyzed the data with the assistance of EH and JW. JW analyzed the monitor data. JW obtained funding and ethical permission to conduct the study. SJ and EJ drafted the first version of the manuscript. All authors contributed to the final version of the manuscript. JW takes responsibility for the manuscript as a whole.

## Funding and Support

This work was supported by 10.13039/100016670Region Östergötland with one grant to author JW (RÖ-979172). Author JW has also received a grant from the regional Lions Clubs International Sweden district. The funding bodies had no role in the design, data collection, analysis, or writing of this study.

## Data Sharing Statement

The monitor alarm data and anonymized interview transcriptions will be made available on reasonable request to the corresponding author.

## Conflict of Interest

All authors have affirmed they have no conflicts of interest to declare.
