# Supplementary material for: Alarm Fatigue in the Emergency Department: A Multicenter, Mixed-Method Study of Monitor Alarms
Source: J Am Coll Emerg Physicians Open. 2025 Feb 27;6(2):100077. doi: 10.1016/j.acepjo.2025.100077 (PMC11919588; doi:10.1016/j.acepjo.2025.100077)
Supplement: Supplementary Material [file mmc1.docx]

# Appendix E1

**Table A1 - Threshold Value that trigger the alarms**

|  |  | **Rural ED** | | | **Academic ED** | | | **Urban ED** | |
| --- | --- | --- | --- | --- | --- | --- | --- | --- | --- |
|  | **Type of monitor** | **MX450** | **MX550** | **X3** | **MX450** | **MX550** | **X3** | **MX450** | **X3** |
| **Alarms** | **Hypoxia(SpO₂)** | **<90%** | **<90%** | **<90%** | **<90%** | **<90%** | **<90%** | **<90%** | **<90%** |
|  | **Heart frequency** | **>130 or <40** | **>130 or <40** | **>130 or <40** | **>130 or <40** | **>130 or <40** | **>130 or <40** | **>150 or <40** | **>130 or <40** |
|  | **Ventricular tachycardia (bpm and runs of ventricular rhythm)** | **>100 bpm and 5 runs** | **>100 bpm and 5 runs** | **>100 bpm and 5 runs** | **>100 bpm and 5 runs** | **>100 bpm and 5 runs** | **>100 bpm and 5 runs** | **>100 bpm and 5 runs** | **>100 bpm and 5 runs** |
|  | **Non-invasive blood pressure (systolic)** | **>180 or <90** | **>180 or <90** | **>200 or <90** | **>180 or <90** | **>180 or <90** | **>180 or <90** | **>180 or <90** | **>180 or <90** |
|  | **Desaturation (SpO₂)** | **<80%** | **<80%** | **<80%** | **<80%** | **<80%** | **<80%** | **<80%** | **<80%** |

note: At the rural ED there was a higher threshold for non-invasive blood pressure when monitored with an X3 model compared with a MX450 or MX550. At the urban ED it was a higher threshold for high heart frequency when monitored with a MX450 compared to X3. The academic ED had the same thresholds regardless of which monitor the patient was monitored with.

**Figure F1: Quantile regression
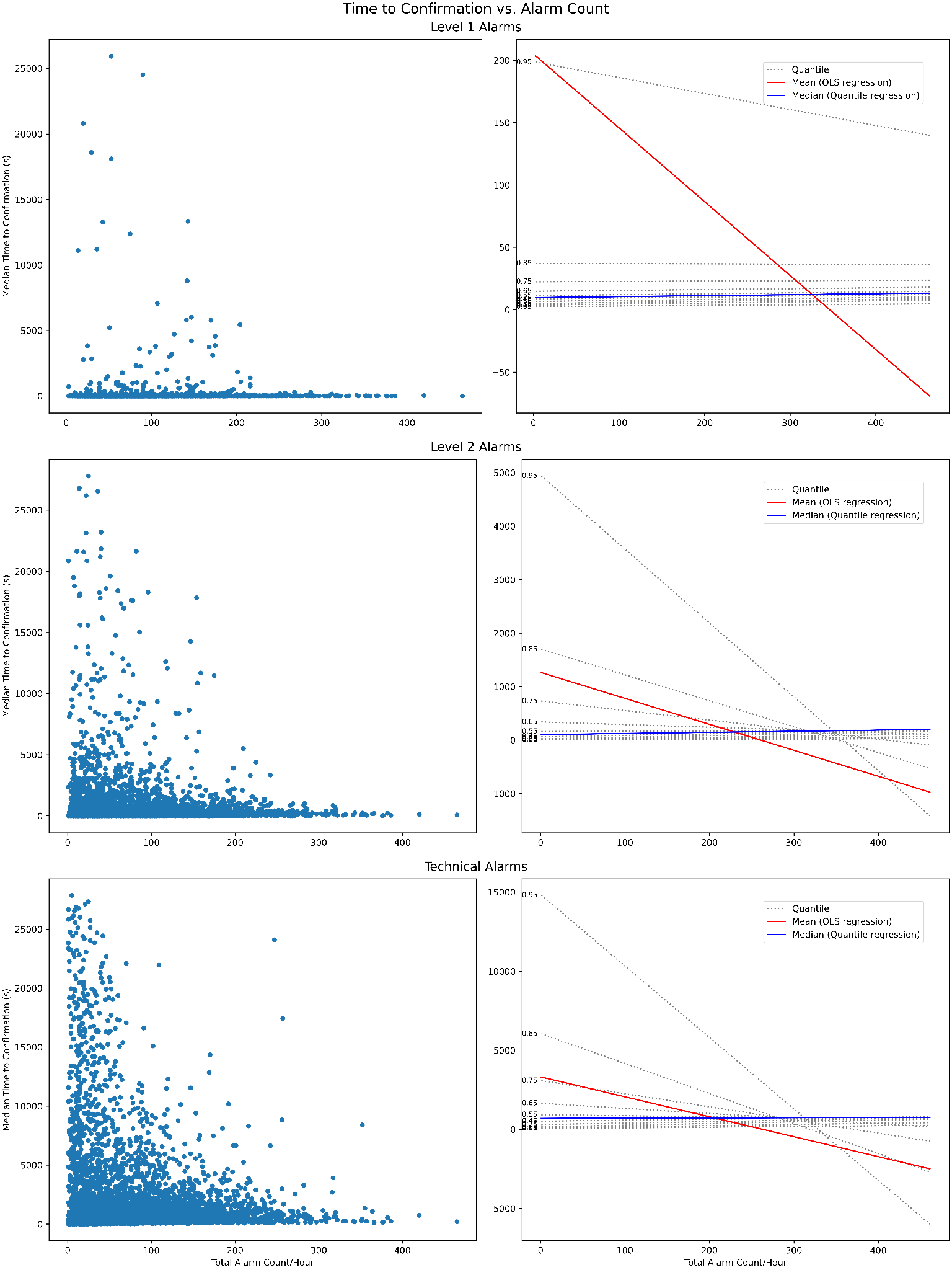
**

Quantile regression on the median confirmation time for each alarm type based on the total number of alarms the same hour. Technical alarms with longer confirmation times than the 90th percentile of length of stay in the included Emergency Departments (8 hours, 28800 seconds) were excluded.

**Table A2: Table of technical alarms**

| Technical alarms | Rural ED | Academic ED | Urban ED |
| --- | --- | --- | --- |
| Number of alarms | 41227 | 147443 | 151291 |
| Unable to read SpO₂ | 15828 (38%) | 38832 (26%) | 59180 (39%) |
| loose electrodes | 8145 (20%) | 32488 (22%) | 26593 (18%) |
| Unable to read respiratory rate | 7683 (19%) | 31532 (21%) | 26045 (17%) |
| Unable to analyze ECG | 4630 (11%) | 19815 (13%) | 24827 (16%) |
| No data on Monitor | 1211 (3%) | 390 (0%) | 4064 (3%) |
| Unable to read NBP | 246 (1%) | 3372 (2%) | 5536 (4%) |
| Other | 3484 (8%) | 21014 (16%) | 5046 (3%) |

Appendix 1 interviewguide

| Question in swedish | Question in english |
| --- | --- |
| Vad tänker du på när vi säger “larm från övervakningsutrustning”? | What do you think of when we say “alarm from monitor equipment”? |
| Kan du berätta om hur du använder övervakningsutrustningen i ditt arbete? | Can you tell us about how you use the monitor equipment in your work? |
| Finns det situationer då du undviker att använda övervakningsutrustningen? | Are there situations where you avoid using the monitor equipment? |
| Berätta vilka faktorer du tror kan påverka antalet larm? | What factors do you think can affect the number of alarms? |
| När på dygnet tror du att det är flest larm? | What time of day do you think there are the most alarms? |
| Hur upplever du att larm från övervakningsutrustningen påverkar ditt arbete på akutmottagningen? | How do you feel that alarms from the monitoring equipment affect your work in the emergency department? |
| Upplever du att vårdpersonal reagerar snabbt på 3-stjärniga larm? | Do you feel that healthcare professionals react quickly to level 1 clinical alarms? |
| Är det något du känner att du skulle vilja lyfta gällande larm kopplat mot ditt arbete på akutmottagningen som vi inte tagit upp? | Is there something you would like to raise regarding alarms linked to your work at the emergency department that we have not covered? |
